# A System of Operative Surgery

**Published:** 1910-09

**Authors:** 


**A System of Operative Surgery.** By Various Authors. Edited by F. F. Burghard, M.S., F.R.C.S. Vol. IV. Pp. xxvi, 687. London: Oxford Medical Publications. 1909.

This last volume of the very handsome and exhaustive treatises on surgical operations deals chiefly with the various special regions. Mr. Bland-Sutton writes eleven chapters on abdominal gynæcology, and gives a great deal of interesting information with regard to pathology, with the clear illustrations which always accompany his writings. Operative methods are relegated to a quite secondary position, as may be judged from the fact that whilst seven pages are devoted to discussing the relations of pregnancy and uterine tumours, less than one page without a single picture is considered sufficient for the description of Wertheim's operation. Dr. Phillips deals with vaginal gynæcological conditions, but there is nothing specially new or original in his article.

The section on ophthalmic surgery, by M. S. Mayou, occupying 125 pages, very fairly represents the practice adopted by English surgeons. When restricted to so limited a space many details have necessarily to be omitted, and the author has acted wisely in leaving out reference to other writers and in reducing to as few lines as possible any criticism of the special advantages and applicability of the operations described. We think, however, that he might have entered rather more into detail in some practical points. Very little is said about the proper application of cocaine, and the duties of the assistant are not clearly defined. In the extraction of cataract an iridectomy is recommended, and capsule forceps are preferred to the cystotome. A much more definite description of the needles and sutures required in advancement operations for strabismus is needed. "Three strong silk sutures" and "four sharp needles" gives no idea whatever to the uninitiated as to what to provide, and the text does not make it very clear what is done with the fourth needle. It is a great advantage to use black silk sutures. Tenotomy of the opposing muscle is advocated in all cases. This no doubt is usually necessary, but there are decided advantages in postponing the tenotomy until the three sutures have been inserted in their places.

In the description of Hess's operation for ptosis the sutures are directed to be passed from the intermarginal line (between the lashes and the border of the conjunctiva) instead of through the skin of the lid at a point some five millimetres above the lashes. This departure from the original description of the operation may be a decided advantage. In describing Motais' operation, no mention is made of the "anchor sutures" fastened above to strapping on the forehead, by means of which the lower lid is raised, and risk of ulceration of the cornea from exposure is to a great extent obviated. Too pessimistic a view, we think, is taken of scleral puncture in detachment of the retina. Deutschmann's and other modifications of scleral puncture are so safe that they are well worth a trial, as extremely good results have been obtained in a few cases.

The descriptions of the operations are very clear and easy to understand. The illustrations are for the most part admirable. Those relating to iridectomy are particularly good, and where necessary pathological details are shown in excellent photo micrographs.

The article on ear operations is by Mr. Hunter Tod, and is a model of clear description, exposition of up-to-date methods, and beautiful illustrations. Mr. Douglas Harmer gives a most complete account of throat surgery, including a description and discussion of bronchoscopy and tracheoscopy. Dr. St. Clair Thomson concludes the volume with the section on nasal operations, those portions dealing with the accessory sinuses being specially good and well illustrated.

